# Digital Inclusion as a Foundation for Health Equity. Comment on “Expanding Video Consultation Services at Pace and Scale in Scotland During the COVID-19 Pandemic: National Mixed Methods Case Study”

**DOI:** 10.2196/34702

**Published:** 2022-02-03

**Authors:** Christopher Weatherburn

**Affiliations:** 1 National Health Service National Services Scotland Edinburgh United Kingdom; 2 National Health Service Tayside Dundee United Kingdom

**Keywords:** technology-enabled care, video consultations, quality improvement, COVID-19, PERCS framework, remote consultation, Scotland, general practice, digital inclusion, digital divide, digital health equity

This insightful paper by Wherton et al [1] suggests groundwork before the pandemic allowed health care services to rapidly extend the use of video consultations across Scotland. As a general practitioner and digital champion, working in a deprived area in a city in Scotland, I agree but strongly believe that front-line staff is fundamental to further increase use. The “asymmetric development, driven in some places by particular local enthusiasts” noted before the pandemic certainly continues, and additional local “champions” are key to further increased use.

The finding relating to the “focus on region-by-region” quality improvement approach noted can be explained by how the National Health Service is set up across Scotland, with 14 different health boards [2]. Although it is pertinent to point out the challenges of providing rural care, most citizens in Scotland live in nonrural areas [3].

My urban general practice embraced the opportunity to perform video consultations. We implemented these by deciding to “convert” telephone consultations to video consultations, when clinically appropriate. This occurred if the patient agreed and had the appropriate technology. We welcomed the improvement allowing the Near Me video service to send direct links to the virtual waiting room.

However, despite this improvement, when discussing video consultations with colleagues, we perceived that many patients then faced technical and accessibility issues. Patients frequently could not enter the virtual waiting room due to low levels of digital literacy.

A recent internal practice audit performed in August 2021 showed that only around half of the patients opted for a video consultation (n=67, 46%) if offered instead of a telephone consultation. Furthermore, of those who requested a video consultation, a large proportion was unsuccessful (n=31, 45%).

If a patient was unable to connect to the video consultation, they would not have the opportunity to complete the patient satisfaction survey, which is given out after the video consultation. Therefore, the patient survey, which reported that the majority of video consultations had no technical problems (n=18,817, 78%), is potentially an underestimate as it does not include some consultations in which patients were unable to access the service at all.

I am pleased to report that the Near Me video service has just been upgraded to include a Consult Now feature. This enables a one-time-only link to be sent, bringing the patient directly to the video call, without having to enter their details and access the virtual waiting room.

In an attempt to offer patients choice, I believe patients should be offered a video consultation instead of a telephone consultation, if they prefer. A video consultation may not objectively aid the consultation; however, it can assist with the clinical relationship.

To do this while addressing digital inclusion, including digital access support is fundamental. We must be mindful to continue to strive to reduce health inequalities and address the “digital inverse care law.” I am personally very supportive of Scotland’s digital health and care strategy, which aims to achieve world-leading levels of digital inclusion [4].
